# The “WEIRDEST” Organizations in the World? Assessing the Lack of Sample Diversity in Organizational Research

**DOI:** 10.1177/01492063241305577

**Published:** 2025-01-22

**Authors:** Robin Schimmelpfennig, Christian Elbæk, Panagiotis Mitkidis, Anisha Singh, Quinetta Roberson

**Affiliations:** University of Lausanne (UNIL); Aarhus University; Aarhus University; London School of Economics and Political Science; Broad College of Business, Michigan State University

**Keywords:** WEIRD, sample diversity, generalizability, systematic review, research methods, theory development

## Abstract

Sampling data from organizations and humans associated with those organizations is essential to organizational research. Much of what we know about organizations is based on such work. However, this empirical foundation may be compromised, calling into question the field’s theoretical and empirical findings. Studies often sample data from relatively similar, narrow contexts, so a lack of sample diversity accumulates in the discipline. To conceptualize this lack of sample diversity and examine its prevalence across research publications, we conduct a pre-registered systematic review of articles from 2018 to 2022 in six top management journals and another systematic review of articles from 2013 to 2022 in six additional journals (not pre-registered). Our review assesses sample country diversity while also exploring within-country factors that are relatively under or oversampled, such as the size or industry of the sampled organization. We find a lack of sample diversity, for instance, a strong bias toward WEIRD (Western, educated, industrialized, rich, and democratic) samples and an underrepresentation of small and medium-sized enterprises in organizational research. Based on the findings and past work, we introduce a conceptual framework for sample diversity along three dimensions: the sample’s geographical, organizational, and personnel contexts. Additionally, we discuss factors that contribute to a lack of sample diversity and propose guidelines for authors, reviewers, and editors to enhance it. Overall, this article seeks to improve the robustness and relevance of theoretical and empirical organizational research, thereby preventing the formulation of misinformed policies and practices in both organizational settings and broader societal contexts.

Given that organizational science is a branch of social science, collecting data from organizations and individuals within them is an essential part of research. Yet, sampling approaches used within the discipline have likely constrained our understanding of organizational phenomena. For example, reviews suggest that the vast majority of research samples, around 70 to 80 percent, are drawn from the United States and Europe (Arnett, 2008; Thalmayer, Toscanelli, & Arnett, 2020), regions that collectively represent only about 11 percent of the world’s population. This heavily skewed sampling toward participants from comparatively WEIRD (Western, educated, industrialized, rich, and democratic) countries and contexts led Henrich, Heine, and Norenzayan (2010) to posit that research in the social sciences merely concerns “the WEIRDEST people in the world.” More specifically, if the empirical foundations of our studies are mainly built from data sampled from WEIRD contexts, our collective understanding may capture only a fraction of human psychological and behavioral variation. This critique of an overreliance on a select few sample contexts has resonated with researchers across the social sciences, including psychology (Arnett, 2008; Thalmayer et al., 2020), economics (Chelwa, 2021), and learning and education (Gaias et al., 2020). With some exceptions (Pitesa & Gelfand, 2023; Zickar & Keith, 2023), organizational research has not systematically assessed sample diversity and its implications for the field.

Our paper addresses this limitation by examining whether research in the discipline primarily draws from a narrow slice of organizational variation, particularly specific origins (e.g., primarily from the United States and Europe), contexts (e.g., industries, firm sizes), or types of participants (e.g., student samples, online panels, managers). Building on prior studies that have explored sample diversity within specific data collection methods (see Porter, Outlaw, Gale, & Cho, 2019) and subfields (see Pitesa & Gelfand, 2023), we systematically review the origin and context of data samples in empirical publications. In doing so, we aim to investigate sample bias within organizational research and articulate its theoretical implications for future work within the discipline.

As the backronym WEIRD was never intended as a theoretical construct but rather as a rhetorical device to draw attention to a pervasive sampling issue (Henrich et al., 2010), we do not attempt to operationalize it directly. Instead, our assessment of sample diversity emphasizes country diversity and other sample characteristics identified in our review to explore the influence of sampling biases on empirical and theoretical findings in organizational research. For example, theories on leadership styles (Lonati, 2020; Lonati & Van Vugt, 2023) or CEO compensation (Tosi & Greckhamer, 2004) are often derived from Western corporate contexts. Consequently, we lack an understanding as to whether the theoretical and empirical findings generalize to other populations, as they may not hold true or be as effective in different cultural settings, raising concerns about internal validity. Similarly, models of organizational change or corporate innovation, predominantly tested in large multinational corporations, may not apply to smaller enterprises or nonprofit organizations that operate under different constraints (Van Essen, Otten, & Carberry, 2015), leading to questions about external validity. These examples highlight a need for more inclusive sampling in organizational research to better understand the boundaries of findings and enhance generalization across different organizational landscapes and contexts (Short, Ketchen, & Palmer, 2002; Stahl, Filatotchev, Ireland, & Miska, 2023). By incorporating more diverse samples, we can strengthen both internal and external validity by ensuring that organizational theories and models are robust, adaptable, and reflective of the varied realities they aim to explain (see Short et al., 2002 for an example of strategic management sampling practices). Importantly, our argument is not that sample diversity guarantees broader theoretical applicability. Rather, the key is ensuring such diversity reflects meaningful variations in the factors shaping organizational phenomena.

Our review is divided into three parts. First, we explore examples from the literature that illustrate how reduced sample diversity can constrain our understanding of the generalizability and applicability of research findings and discuss the importance of sample diversity for theory building. Second, we present a pre-registered (see, https://osf.io/7s4ye) systematic review of research samples from articles published between 2018 and 2022 in six top-tier management journals: *Academy of Management Journal*, *Administrative Science Quarterly*, *Journal of Management*, *Management Science*, *Organization Science*, and *Strategic Management Journal*. To widen the scope of the review and increase the robustness of its conclusions, we also present exploratory findings from six additional journals in the field. Drawing on these findings, we introduce a conceptual framework for categorizing sample diversity, which incorporates samples’ geographical, organizational, and personnel contexts and offers a structured approach for future organizational research. Third, we highlight key insights from our review and offer guidelines for authors, reviewers, and editors to enhance sample diversity within the field.

Our article makes several contributions. We advance an understanding of the importance of sample diversity in organizational research methods, highlighting its critical role in shaping the accuracy and relevance of empirical findings. By exploring the effects of contextual variation within the field, we theorize how sampling biases may constrain inductive precision and limit the scope of theory in organizational research. We also extend the conceptualization of diversity by systematically reviewing sample diversity across geographical, organizational, and personnel contexts. This allows us to broaden the theoretical conversation on diversity, moving beyond individual-level attributes to consider how contextual factors influence the generalizability of research findings and the replicability of our science. In effect, we offer a novel framework that positions diversity as a crucial variable for assessing the robustness and applicability of organizational theories. Finally, we offer future directions for authors and editorial teams to enhance sampling diversity, thereby reinforcing the conceptual foundations of organizational science. By promoting more inclusive and nuanced theories that reflect the complexity of organizational behavior across different settings, industries, and cultural contexts, we contribute to the advancement of theory-building and theory-testing within the discipline.

## Sampling Constraints in Organizational Research

Sample diversity in organizational research refers to the extent to which the empirical foundation covers a wide range of samples that reflect diverse organizational contexts and settings encountered in practice (Henrich et al., 2010; Rosenzweig, 1994; Short et al., 2002). An issue arises when samples for theory testing are not drawn from the wide variety of available contexts. For example, organizational studies often lack sample diversity when researchers rely on publicly available databases, existing links with organizations or managers, or more readily obtainable student, online, or convenience samples (Peterson & Merunka, 2014; Short et al., 2002). In other instances, sample diversity is constrained by targeted homogenous sampling from a specific context rather than limits on availability. While both can be suitable for answering particular research questions, they contribute to an overall lack of diversity in the discipline’s samples. Often, this gap involves underrepresented contexts, such as organizations unwilling to share internal data or those in less developed countries with data infrastructure. As a result, variation in organizational practices and the human experience is systematically overlooked.

The criteria for determining when and how generalizable conclusions can be drawn remains a subject of debate across disciplines (Rosenzweig, 1994; Short et al., 2002). As researchers, our ability to extend findings to broader contexts depends upon the data from which those findings are derived. For example, in a replication of a study examining priming effects on dishonesty among Swiss bankers, the results revealed potential limits in generalizability to the Middle East and Asia Pacific regions (Rahwan, Yoeli, & Fasolo, 2019). More specifically, after controlling for identity and geographic culture effects, variations across banking segments and institutional norms produced differences in honesty effects. Similarly, Medvedev, Davenport, Talhelm, and Li (2024) found stronger motivating effects of monetary versus psychological incentives in WEIRD samples, particularly drawn from the United States and United Kingdom, compared to those from China, India, and South Africa. Research also shows that the effects of organizational phenomena fluctuate based on contextual factors at lower levels of analysis. In particular, studies have shown that industry context, occupational demography, and organizational size affect relationship strength (Jacobs & Watts, 2021; Joshi & Roh, 2009). Additionally, other diversity-related factors, such as average employee age and tenure, have been shown to impact effect sizes within organizational research (Dobrow, Ganzach, & Liu, 2018). For an overview, see Online Appendix 1. Consequently, the ecological validity of organizational science—largely conducted within WEIRD contexts—may be called into question.

Sampling choices are typically considered to be a methodological issue, but they also constrain our ability to test, refine, and build theories that are applicable across diverse contexts. Theories developed and tested using more homogeneous subpopulations that are less representative of the larger context may lead to a narrow and potentially biased understanding of phenomena (Peterson & Merunka, 2014; Short et al., 2002), particularly regarding their boundary conditions (Busse, Kach, & Wagner, 2017). Moreover, a lack of variation among those who develop and publish these theories can skew the types of theories that are prioritized and explored (Al-Aiban & Pearce, 1993; Kiggundu, Jørgensen, & Hafsi, 1983). In other words, sample diversity is critical throughout the entire theory-building process.

Theory building, as an iterative but also path-dependent process, often begins with the identification of phenomena and initial observations, which may be gathered through case studies, direct observation, or surveys (Eisenhardt & Graebner, 2007). This initial process is typically shaped by the context and environment in which the researchers operate. In simpler terms, research ideas often emerge from personal experiences or topics researchers have previously experienced or explored. When this holds true, the initial identification of the studied phenomenon and the early observations will be influenced by the authors’ contexts and sample characteristics. This initial contextual bias can then carry through to subsequent stages of theory building, particularly in hypothesis testing and refinement, as theories and hypotheses are tested and refined in other samples (Langley, 1999).

For example, Tosi and Greckhamer (2004: 1) critique CEO compensation theory and research as “dominated by assumptions and values reflective of those dominant in the national culture of the United States,” emphasizing its focus on *competition and capitalism. They also* highlight the limitations of relying on economic theories that are rooted in individualistic Western values, such as agency and tournament theory, to explain CEO compensation as a tool for maximizing performance within corporate governance frameworks. Similarly, although leadership occurs across a variety of organizational contexts—ranging from S&P 500 companies to small- and medium-sized enterprises (SMEs) and non-governmental organizations (NGOs)—much of leadership theory has been developed by prototypical leaders within the United States (Lonati & Van Vugt, 2023). Accordingly, leadership theory is primarily reflective of Western perspectives, overlooking the diverse leadership approaches and processes found in other parts of the world (Garfield & Hagen, 2020; Sanchez-Runde, Nardon, & Steers, 2011). As a lack of variation in the home countries of scholars who formulate and publish these theories can further distort our understanding of leadership emergence and functioning, a more inclusive approach to sampling within leadership and other areas of organizational research is essential for developing more comprehensive and universally applicable theories.

In general, reliance on homogeneous samples, whether due to convenience or targeted sampling, leads to a narrow understanding of organizational phenomena and neglects the rich diversity present in organizational contexts. This limitation compromises the generalizability of research findings and hampers the refinement and testing of global theories of work. The dominance of WEIRD perspectives can alter our understanding and obscure valuable insights from other cultural and organizational contexts. Therefore, a concerted effort to incorporate more diverse samples in organizational research is essential for enhancing the ecological validity of our science and ensuring that our theoretical frameworks are robust and inclusive. Broadening our sampling practices allows us to test for assumptions embedded in our theories and research and understand their applicability across different populations. As sample diversity is not merely a matter of enhancing generalizability, adopting a less WEIRD approach to organizational research is also fundamental to constructing theories that authentically capture the heterogeneity and complexity of organizational life. Below, we present our systematic review of organizational research published between 2018 and 2022 to examine various forms of sampling bias and develop a framework for enhancing sample diversity in future work.

## Review of Sample Diversity in Organizational Research

Systematic assessments of sample diversity and initiatives to diversify research samples within psychological research have increased over the past years (e.g., Adetula, Forscher, Basnight-Brown, Azouaghe, & IJzerman, 2022; Arnett, 2008; Ghai, 2021; Thalmayer et al., 2020). For example, the Psychological Science Accelerator (https://psysciacc.org/), a consortium that unites teams of scientists from a pool of thousands of researchers located in over 70 countries to facilitate global research collaborations, has been effective in generating greater sample diversity. Yet, as psychological research predominantly samples human subjects, often in university labs or via online panels, sampling within the organizational sciences may be qualitatively different. More specifically, as organizational research often samples at the organization level of analysis and utilizes secondary data, such as patents and historical GDP records, a systematic review of the management literature may be needed to understand the nuances of sampling bias within the field.

Extant reviews on sample methodologies have primarily focused on specific sampling methods or participant types within the organizational literature (see Boussebaa, 2024; Pitesa & Gelfand, 2023; Zickar & Keith, 2023), such as the selection of sampling techniques (e.g., convenience or stratified sampling), data collection types (e.g., observational or survey data), and sample participants (e.g., undergraduate students or employees at an organization). Recent reviews have also analyzed the reliance on online panel data (Aguinis, Villamor, & Ramani, 2021; Porter et al., 2019), student subject pools (Kees, Berry, Burton, & Sheehan, 2017; Peterson & Merunka, 2014), and convenience samples (Short et al., 2002). We build on these findings by considering the full spectrum of sampling methodologies employed across organizational research disciplines and within both micro and macro domains.

Informed by discussions in diversity research (Bell, Villado, Lukasik, Belau, & Briggs, 2011; Harrison & Klein, 2007; Joshi & Roh, 2009; Roberson, 2019), we appreciate that sample diversity in organizational research is multidimensional. Accordingly, we draw on the WEIRD concept (Henrich et al., 2010) and past approaches to sample diversity in other disciplines (Arnett, 2008; Chelwa, 2021; Linxen, Sturm, Brühlmann, Cassau, Opwis, & Reinecke, 2021; Thalmayer et al., 2020) as our starting point and assess sample diversity at the country level. In the quest for generalizability, we agree that country-level generalizability is often not the single best way to gauge diversity and identify a level of analysis, for example, to identify the borders of a cultural group. The country level might be both too broad and too narrow to classify culture (Schimmelpfennig et al., 2024a, 2024b). While we report some clustering of cultures on the country level, we also recognize that socioeconomic status can be a more significant driver of similarity than geography (White & Muthukrishna, 2023). In addition, there were practical reasons to use the sample’s country of origin as an identifier, as it is one of the few sample pieces of information often available across the papers sampled. However, we also explored sample diversity beyond the country level. We used a large language model (LLM) to examine sample diversity more granularly, including industry, organization size, and participant type, to determine representation within the literature. By adopting this broader lens, our review highlights the nuances and complexities of sampling in organizational research that have not been addressed in previous works.

### Conducting Our Systematic Literature Review

Our pre-registered literature search (see https://osf.io/7s4ye), conducted in the Web of Science database, includes all empirical articles published between 2018–2022 in six top-tier management journals with a focus on impact factors and empirical research (i.e., *Academy of Management Journal*, *Administrative Science Quarterly*, *Journal of Management*, *Management Science*, *Organization Science*, and *Strategic Management Journal*). While all of these journals are based in the United States, they do not limit their scholarship to the United States and represent diverse fields of organizational research. Accordingly, our selection criteria capture a wide spectrum of research methodologies, theoretical approaches, and empirical findings. The five-year period was chosen to get a representative overview of recent literature in organizational research and follow conventions in other disciplines (Arnett, 2008; Thalmayer et al., 2020). The initial search, excluding commentaries, book reviews, and editorials, yielded a total sample of *N* = 3,969 articles. Consistent with the approach used by other researchers (Antonakis, Bendahan, Jacquart, & Lalive, 2010; Bergh, Perry, & Hanke, 2006; Fischer, Dietz, & Antonakis, 2017), we randomly selected 2000 articles from the initial population to evaluate a more manageable yet representative subsample.

Two coders were trained to code the articles. The first one coded for a pilot sample of 100 articles (PaperIDs 1–100), which allowed us to calibrate the coding framework. After the pilot coding, we preregistered the inclusion criteria and coding framework for the systematic review (link to pre-registration, data, and scripts for analysis: https://osf.io/auszy/). These initial 100 articles were omitted from the full review and the first coder coded for the remaining 1,899 articles (PaperID 101-1999). In parallel, a second coder coded for 500 articles (PaperID 101-600). To ensure coding reliability, we calculated inter-coder reliability for the two main coding events per article (i.e., the variable *Research Type*, which determined whether an article included empirical work and therefore was or included in the review, and *Sample Country*, which had information about where the data had been sampled). The two coders agreed in 84% (Research Type) and 86% (Sample Country) of the cases. We calculated the inter-coder reliability, which produced Kappa values of κ = .86 and κ = .86, respectively, and resolved all disagreements between the two coders. Further, only the first coder coded the total sample of articles.

To enhance the breadth and robustness of our findings, we coded an additional 500 articles from six different journals in organizational research (exploratory analysis not pre-registered). To ease the description, we will refer to this set of journals as the “additional journals,” and the initially (and pre-registered) set of journals as the “baseline journals.” The literature search for the coding of the articles from the additional journals was also conducted in the Web of Science database. We include all empirical articles published between 2013 and 2022 in six management journals (i.e., *Journal of Applied Psychology*, *Journal of Management Studies*, *Journal of International Business Research*, *Human Resource Management*, *Journal of Organizational Behavior*, and *Human Relations*). These journals broaden the scope of our initial systematic review regarding topics, research methodologies, theoretical approaches, and time. A more extended period (10 years in the additional journals instead of 5 years in the baseline journals) was chosen to provide a better overview of possible developments over the next 10 years. This review of the additional journals was not part of our initial pre-registration, as this additional coding was suggested in the review process, and we want to thank the reviewers for their recommendation to include these additional journals.

The initial search, excluding commentaries, book reviews, and editorials, yielded a total sample of *N* = 4,078 articles, of which we randomly selected 500 for the coding. The same two coders who had coded the articles in the baseline journal review again coded these articles to maintain consistency. Each of the coders coded 250 articles. The complete codebook and coded articles can be found in the online appendix: https://osf.io/j6fu5?view_only=None.

### Insights from the Systematic Review

Among the fully coded 1899 articles in the baseline journals (PaperIDs 101-1999), 1,259 (67%) articles were quantitative and 137 (7%) were qualitative empirical work; 61 (3%) were coded as mixed methods, while the remaining 442 (23%) articles were classified as non-empirical and, thus, excluded from further review (see Table 1). Moreover, of the 1457 empirical articles, 363 (25 %) used primary data collection (e.g., surveys or laboratory and field experiments), 933 (64%) used secondary data collection, and the remaining 161 (11%) used both primary and secondary data collection. Interestingly, 506 articles (35% of all empirical articles) sampled human participants, a notable difference from research in psychology and organizational behavior, which almost exclusively sample human participants. This confirms our initial motivation to understand sample diversity across different data collection methods.

**Table 1:** Summary Statistics of Systematic Review for the Total Sample of Reviewed Articles and by Journal

Journal	Article Count	Research Type				Data Collection			Human Participants Sampled	
		Quantitative	Qualitative	Non-Empirical	Mixed Methods	Primary Data	Secondary Data	Both Primary and Secondary	No Human Participants	Human Participants
*AMJ*	167	123	36	1	7	64	69	33	70	96
*ASQ*	66	24	30	2	10	16	27	21	27	37
*JOM*	215	146	12	54	3	80	66	15	67	94
*MS*	963	631	1	327	4	132	486	18	498	138
*OS*	225	139	45	17	24	51	104	53	108	100
*SMJ*	263	196	13	41	13	20	181	21	181	41
Total	1,899	1,259	137	442	61	363	933	161	951	506

*Note.* AMJ: *Academy of Management Journal*, ASQ: *Administrative Science Quarterly*, JOM: *Journal of Management*, MS: *Management Science*, OS: *Organization Science*, SMJ: *Strategic Management Journal*

#### Sample diversity: variation of sample countries

We first coded for the origin country and region of the sample used in each study. To code for the sample country of origin, the coders read through the abstracts, introduction, and methods sections until the information was found. In some cases, we also had to review the articles’ appendices or supplementary information to determine the sample country and region. If samples were reported to originate from several countries, the article was coded as “*International*.” If we could not infer the sample origin beyond a reasonable doubt, we coded the sample country as “*notgiven*” (see Online Appendix 2 for relative frequencies of “*International*” and “*notgiven*”; https://osf.io/j6fu5?view_only=None).

**Figure 1:**
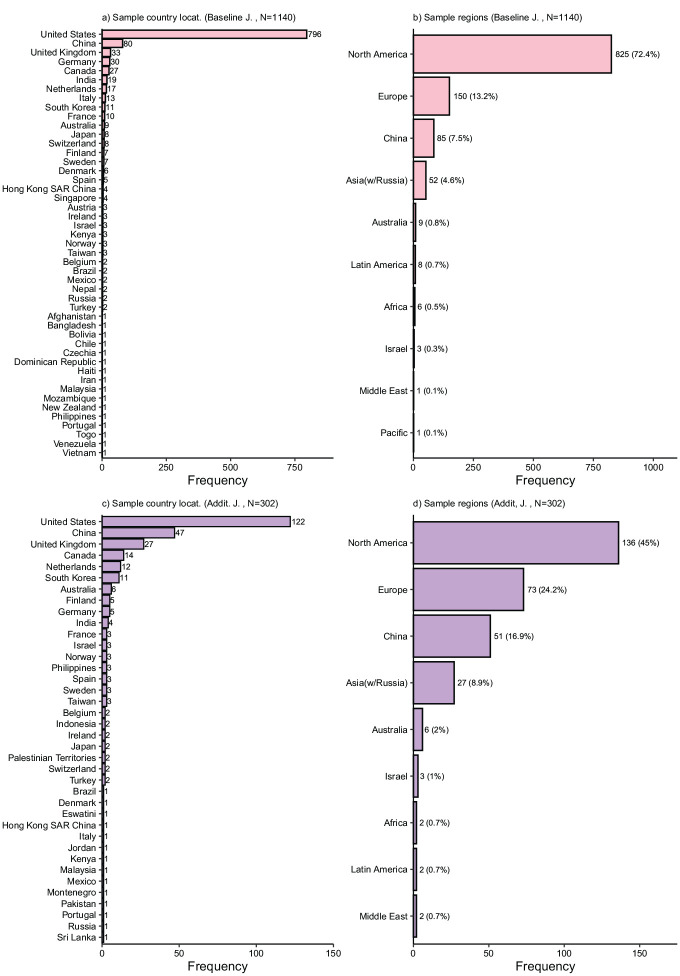
Sample Origin by Country and Region *Note.* Panel a (baseline journals) and panel c (additional journals) show the frequency of countries from which articles were sampled. Panel b (baseline) and panel d (additional) show the frequency of regions from which articles were sampled. In this overview, we excluded non-empirical work and articles without information about a specific sample country.

Figure 1 summarizes the distribution of sample countries and regions with baseline journals shown in panels (a) and (b) and additional journals in panels (c) and (d). The figure reflects articles in which we identified a single sample country.

The plotted frequencies reveal a strong skew in sample locations. Analyzing the results for the baseline journals, the overwhelming majority of samples originate from the United States, as those samples are nine times more frequent than the next-biggest sample origin country, China. Further, whereas samples from several European (e.g., Germany, United Kingdom, Netherlands, Italy) and Asian (South Korea, India, and Singapore) countries were present among the reviewed articles, almost no samples originated from countries in Africa, Latin America, or the Middle East. While each region has a larger population than North America (in our definition, including the United States and Canada), their combined relative frequency was around 1% of all empirical articles. Altogether, 86% of all articles for which we identified a single country derived its sample from a North American, European, or Australian (i.e., a “Western”) context. In the additional journals, the share of North American and European samples is smaller (71.2% from the United States, Europe, or Australia), explained by an increase in samples from Asian countries. The combined share of samples from Africa, the Middle East, and Latin America is only around 2%.

#### Mapping geographical diversity onto relevant country-level contexts

**Figure 2:**
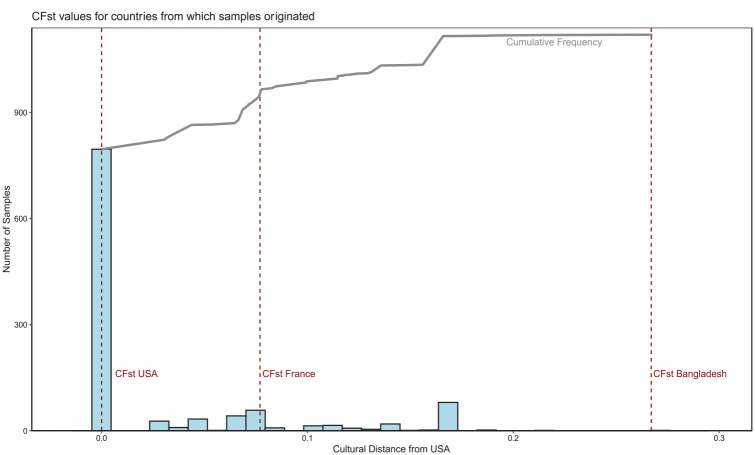
Distribution of CFST Values of Sample Countries *Note.* The figure shows a lack of cultural diversity in the samples. We graph the distribution of CFST (cultural distance from the United States) values (Muthukrishna et al., 2020) for sample countries from the baseline journals that are included in the World Value Survey. The dashed line on the right represents the CFST value for France, a relatively WEIRD country. The dark gray, solid line indicates the cumulative frequency over increasing CFST values. This shows that few samples are more culturally distant from the United States than France (e.g., China is the country with the largest cultural distance to the United States in our sample), indicating a high degree of cultural homogeneity.

To explore the influence of geographic variation on organizational variation, we also examined *culture* as a contextual factor, given that it has been shown to vary across countries and to impact the creation and implementation of organizational practices (Gelfand, Erez, & Aycan, 2007). Culture, defined as the shared set of beliefs, norms, skills, and practices in a given context, can produce significant differences in psychological and behavioral outcomes (Gelfand et al., 2011; Muthukrishna et al., 2020) and different ways of organizing (Talhelm et al., 2014). Accordingly, we allocated sample countries with a value for Cultural Fixation (*CF_ST_*), an empirically informed, continuous measure of cultural differences between populations over various social, psychological, and cultural traits (Muthukrishna et al., 2020). The *CF_ST_* score is based on hundreds of thousands of responses in the World Values Survey (Inglehart et al., 2014), which spans over 60 countries and has been used to calculate a country’s cultural distance from the United States as an index to gauge how WEIRD a specific sample is (Muthukrishna et al., 2020). In line with previous studies and because most samples in our review originate from the United States, we use this cultural distance, which can theoretically range from 0 (in the United States) to 1, as a continuous indicator of a country’s “WEIRDness.” Figure 2 shows the frequency distribution of samples from the baseline journals across different *CF_ST_* scores. Only 16% of samples are culturally more distant from the United States than France, a country many consider WEIRD. This indicates a high degree of cultural homogeneity across samples relative to the amount of cultural heterogeneity in the world. It is important to note that the results of reduced sample diversity were the same for other contextual factors relevant to the organizational landscape of a country, such as economic development (GDP), industrialization (e.g., Industrial Development Report: United Nations Industrial Development Organization, 2015), digitalization (Internet Penetration: World Bank Group, 2017), and property rights (World Bank Group, 2020). See Online Appendix 3 for an overview.

### Sample Diversity Beyond the Country Level

To further explore emerging aspects of sample diversity beyond the country level, we conducted an exploratory analysis using the LLM GPT- 4 Turbo via the OpenAI-API to assist in coding reviewed articles. Our process began with preparing structured prompts, in which we prompted the model to iteratively go through all empirical articles that used primary data (*N* = 524). This procedure allowed us to systematically process and analyze large volumes of textual data efficiently and accurately. For the input prompt, we provided the article’s title and abstract and manually retrieved method and sample descriptions in the reviewed articles. The model’s responses were utilized to code and classify two relevant variables: *organization size* (e.g., startup, SME, multinational organization) and the *industr*y (e.g., real estate, finance), and one relevant variable for the type of *participants* (e.g., online panel data or organizational employees). To avoid a large share of false positive coding, we did not code for a given variable if the information was insufficient to code beyond a reasonable doubt but also when the sampled data did not fit the predefined categories. For example, we could not code for the variable *industry* when the data did not come from an organization (e.g., if an article used the stock performance of Fortune 500 companies). We repeated this automized coding over three repetitions for each article and only retained the modal coding by the model. There was no case where all three repetitions led to a different code, indicating good reliability of the model’s coding and the methodological approach more broadly. As a further indication of the external reliability of this approach, the categorization of the papers from the additional journals (*N* = 271) with the same prompt showed many similarities to the distribution of categories in the baseline journals. For more information, see Online Appendix 4 and the documentation code.

**Figure 3:**
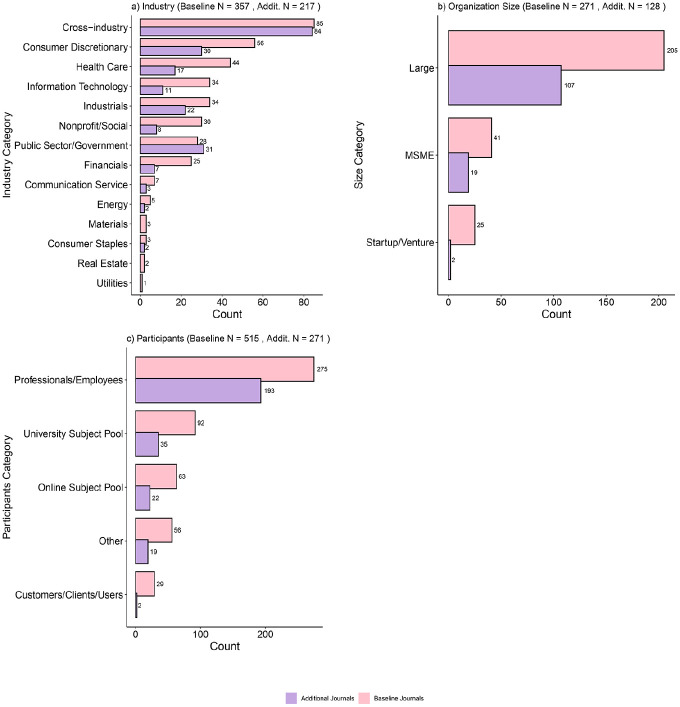
Cross-Contextual Variation *Note.* Cross-contextual variation for (a) industry and (b) organization size, and (c) participants among articles that collected primary data in the baseline journals (light blue, *N* = 524), and the additional journals (red, *N* = 272).

Figure 3 depicts the findings of this sample categorization for both the baseline journals and the additional journals. We can observe a moderate level of variation in industry contexts (panel a). Although the samples were not concentrated in a small subset of industries, grouping industries by the Global Industry Classification Standard (MSCI, 2024) revealed that there are industries with a more significant concentration of samples (e.g., consumer discretionary and healthcare). Among the articles from the additional journals, the government and nonprofit sector also represented a sector often sampled from. In contrast, others remain relatively under-sampled (e.g., energy, utilities, real estate, materials). Although we found that a relatively large share of reviewed articles used cross-industry data to understand cross-contextual variation, closer inspection revealed that these were predominantly from the same indices (e.g., S&P 500). We also assessed the diversity in organization size and found that most data came from large or multinational organizations (panel b). Small and medium-sized enterprises (SMEs) were not often sampled despite their relative frequency in numbers and contributions to overall economic and organizational output (World Bank, 2019; World Trade Organization, 2016).

We also considered sample diversity at the participant level across all articles that used primary data collection (e.g., surveys or experiments). As shown in panel c, we found that samples tended to have a large share of participants sampled in organizational contexts (e.g., professionals and employees). Furthermore, the share of online panel data or university subject pools was relatively small compared to psychological or behavioral science samples and studies within the management subfield of organizational behavior (Porter et al., 2019; Zickar & Keith, 2023). Notably, participants on the demand side (i.e., customers, clients, users) represented the smallest category.

Overall, the distribution of the sampled categories for all three contextual variables is skewed (Figure 3). Yet, we observe at least a moderate level of diversity, which was higher than the skew in the geographical context (Figure 1). These patterns serve to highlight the WEIRDness of samples within organizational research.

### Sample Country Diversity Across Time and Fields

To get a broader view of sample country diversity over time and within specific subject areas and journals, we explored the share of samples from Western contexts (i.e., samples from the United States, Europe, or Australia) across the six baseline and the six additional journals. Overall, we did observe a small decrease in articles with Western samples over time (see Online Appendix 5). In articles from the baseline journals, the average share of Western samples was 86%, with the share ranging from 80% to 89% between 2018 and 2022. For the additional six journals, the overall average was 71%, with a low of 61.3% in 2018 and a high of 82.5% in 2021, thus showing a slightly lower representation of Western samples in the additional journals. To explore this variation in more detail, we recorded the shares of Western samples and their respective subregions across the 12 journals (see Table 2). Only three journals (*Journal of Organizational Behavior*, *Human Resource Management*, *Journal of International Business Studies*) had less than 75% of articles with samples from outside North America, Europe, or Australia, which is largely driven by samples from China and other Asian countries. Consistent with our primary sample, our coding revealed the relative absence of samples from Latin America, Africa, or the Middle East.

**Table 2:** Shares of Samples From Each Region Across Journals

Journal	Count	“Western”	North America	Europe	Australia	China	Asia (w/Russia)	Latin America	Middle East	Pacific	Africa	Israel
***AMJ***	**134**	**0.806**	**0.657**	**0.134**	**0.015**	**0.112**	**0.060**	**0.007**	**0.007**	**0.000**	**0.007**	**0.000**
***ASQ***	**52**	**0.827**	**0.654**	**0.154**	**0.019**	**0.077**	**0.058**	**0.019**	**0.000**	**0.000**	**0.019**	**0.000**
***JOM***	**130**	**0.792**	**0.569**	**0.223**	**0.000**	**0.131**	**0.054**	**0.000**	**0.000**	**0.008**	**0.000**	**0.015**
***MS***	**511**	**0.885**	**0.769**	**0.104**	**0.012**	**0.065**	**0.037**	**0.008**	**0.000**	**0.000**	**0.004**	**0.002**
***OS***	**158**	**0.892**	**0.722**	**0.171**	**0.000**	**0.051**	**0.032**	**0.013**	**0.000**	**0.000**	**0.013**	**0.000**
***SMJ***	**155**	**0.884**	**0.787**	**0.097**	**0.000**	**0.052**	**0.065**	**0.000**	**0.000**	**0.000**	**0.000**	**0.000**
*JMS*	34	0.824	0.500	0.265	0.059	0.088	0.059	0.000	0.000	0.000	0.029	0.000
*HR*	66	0.818	0.273	0.515	0.030	0.091	0.045	0.000	0.030	0.000	0.000	0.015
*HRM*	40	0.575	0.350	0.200	0.025	0.200	0.225	0.000	0.000	0.000	0.000	0.000
*JOB*	50	0.700	0.420	0.260	0.020	0.140	0.100	0.020	0.000	0.000	0.020	0.020
*JIBS*	29	0.414	0.345	0.069	0.000	0.345	0.207	0.034	0.000	0.000	0.000	0.000
*JoAP*	83	0.759	0.675	0.084	0.000	0.205	0.024	0.000	0.000	0.000	0.000	0.012

*Note.* The table only lists articles for which a sample country could be identified. AMJ: *Academy of Management Journal*, ASQ: *Administrative Science Quarterly*, JOM: *Journal of Management*, MS: *Management Science*, OS: *Organization Science*, SMJ: *Strategic Management Journal*, JMS: *Journal of Management Studies*, HR: *Human Relations*, HRM: *Human Resource Management*, JOB: *Journal of Organizational Behavior*, JIBS: *Journal of International Business Studies*, JoAP: *Journal of Applied Psychology*. Shares of samples from each region across journals. The bolded entries mark shares from the baseline journals, and the non-bolded entries mark shares from the additionally coded journals. “Western” samples are samples that originate from North America (the United States and Canada), Europe, or Australia. The categorization of regions is based on previous work (Arnett, 2008). “North America” includes the United States and Canada; “Latin America” includes countries in Central and South America and the Caribbean.

While there is no clear systematic difference in the proportion of Western samples between the two sampling approaches (i.e., both feature journals where Western samples make up over 80%), there are some noteworthy differences that warrant further explanation. For example, the *Journal of International Business* has one of the lowest proportions of Western samples, which is not surprising given the journal’s focus on international sampling. However, a closer look at Table 2 reveals that this international sampling is not evenly distributed across the globe as Latin America, the Middle East, and Africa (home to around 2.5 billion people) are almost completely ignored. On the other hand, the lower proportion of Western samples in the journal *Human Resource Management* is more surprising. Although it is difficult to assess why this is the case without detailed information about the journal’s practices, submissions, and rejections, its website states that the journal “welcomes submissions from across the globe” and had issued a call for papers for submission with a focus on China/Chinese—a language that is missing for many other journals. Is inclusive language alone enough to encourage more diverse sampling? Perhaps not. However, it seems that authors tend to sample where they are based. When journals actively support submissions from a more diverse group of authors, they also may see more diversity in their samples.

## Discussion

Our systematic review, which explores the lack of sample diversity in organizational research, reinforces the idea that research samples within our science are drawn from a relatively thin slice of organizational variation. Given the implications of these findings for the applicability and generalizability of our theories and empirical research, we propose future directions for incorporating greater sample diversity and enhancing the field’s inductive capabilities. We first introduce a sample diversity framework that considers a broader range of contextual factors beyond culture that shape organizational experiences. We outline how researchers can apply this framework to support and enrich theoretical development and strengthen inferences drawn about phenomena across contexts. We also discuss the drivers of sampling biases and offer guidelines for authors and editorial teams to promote sample diversity within the field.

### A Framework for Sampling Across Varied Organizational Landscapes

**Figure 4:**
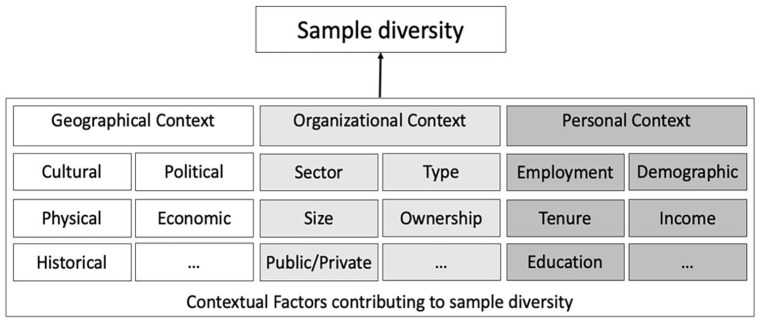
Framework of Sample Diversity *Note.* Overview of three types of contexts that together form a framework of sample diversity. The framework is developed using a two-pronged approach. First, we draw on our review and the summary of critical cross-context factors identified in the review. Second, we integrate the poly-contextual framework of Tsui et al. (2007), research of relevant organizational contexts, and past work on individual- and group-level factors into our framework of sample diversity.

Consistent with cross-cultural research, we examined and found evidence of variation in sample countries. However, our review revealed sampling biases based on other features of context. Specifically, we identified diversity in samples based on geographical, organizational, and personnel factors, which exist alongside one another yet interact to shape complex sample contexts. Building on the work of Tsui, Nifadkar, and Ou (2007), which calls for research that incorporates multiple contexts (e.g., historical, economic, political) to understand the culture and how phenomena operate within it, we argue that organizational research must account for factors within geographic as well as organizational and personnel contexts to generate deeper insights into organizational structures and behaviors. Our framework for sample diversity, presented in Figure 4, details these factors. Below, we discuss the impact of these contextual influences on sample diversity and how researchers can incorporate them into their work.

#### Geographical context

Geographical context refers to the extent to which research samples capture the diverse economic, cultural, and geopolitical environments in which organizations operate. With the majority of the world’s population living outside North America or Europe, and with Asian and African countries experiencing the highest population growth rates, it is essential to ensure that research samples are representative of organizations in an increasingly globalized world (Stanley, 2023). The growing prominence of populations from less WEIRD regions is likely to increase the diversity of the global organizational landscape further, as countries such as India and Nigeria follow China’s economic growth and the institutionalization of organizational activities. Further, while many of the world’s largest firms are still based in North America or Europe, an increasing number are emerging from other regions (PricewaterhouseCoopers, 2022). For example, 20 of the 50 largest companies by revenue come from outside these traditional economic centers (PricewaterhouseCoopers, 2022). This highlights that a significant portion of global economic and organizational activity is occurring in diverse countries. Given projected population trends, the influence and importance of regions outside North America and Europe are only expected to grow further.

Researchers should not assume that low variation in sample countries necessarily represents a research limitation, given that national borders do not always produce variation in organizational practices. The issue arises when the lack of country variation corresponds to limited diversity in factors influencing the antecedents, processes, or consequences of organizational phenomena. For example, cultural factors within a geographical context can drive differences in leadership behavior and preferences (Lonati, 2020), cooperativeness (Schulz, Bahrami-Rad, Beauchamp, & Henrich, 2019), and organizational behavior more broadly (Gelfand et al., 2007; Schimmelpfennig & Muthukrishna, 2023). Because organizations and people within organizations are often culturally and contextually embedded, theory testing that relies on samples from specific cultures and contexts may lead to limitations in understanding the broader applicability of organizational phenomena.

Beyond intentionally including samples from Africa, Latin America, and other less WEIRD contexts to reflect the variation in organizational practices and behavior, future research should consider other geographical features. For example, as organizational structures and strategies are influenced by economic factors, such as industrialization and market dynamics, incorporating these and other aspects of economic diversity into organizational research can deepen our understanding of macro phenomena across different regions. Similarly, as political factors, such as power distribution and political stability, may affect managers’ ability to influence earnings, accounting for these variables may offer valuable insights into the complexities of agency, leadership, and other aspects of organizational behavior. Moreover, the development of theories that address the impact of global events and external shocks on organizational resilience and adaptability can enhance the relevance and contextual richness of our work, offering a better understanding of how organizations can navigate complex, dynamic, and often unpredictable environments. Overall, broadening the geographical scope of research can help to identify both universal principles and context-specific variations in our findings, thereby enhancing the robustness of our theoretical models.

#### Organizational context

As organizational activity spans 11 sectors, 25 industry groups, 75 industries, and 163 subindustries (MSCI, 2024), organizational context captures the representation of various organizational types in research samples. Despite this diversity, much organizational research concentrates on more prominent or accessible sectors and often relies on indices that group organizations from multiple sectors, such as the S&P 500 or Fortune 500. However, these predominantly include organizations from North America, overlooking the intersectionality of geographical and organizational contexts. Additionally, these indices almost exclusively include large, for-profit corporations, which are considered to be the most relevant actors, driving economic growth and employing the most people. While this may be true to some extent, it disregards the critical role of small and medium-sized enterprises (SMEs), which make up the vast majority of businesses worldwide. According to the World Trade Organization (2016) and the World Bank (2019), SMEs constitute more than 90% of all businesses, account for over 50% of employment, and contribute over 50% of gross domestic product (GDP)—reaching up to 40% in emerging economies. These numbers do not include informal SMEs, which would significantly increase their share and economic importance in many emerging markets. Furthermore, economic and technological development, along with increased globalization, continues to diversify the organizational landscape within relatively WEIRD societies. Mid-sized organizations from around the world are increasingly integrated into global supply chains. For instance, in 2017, approximately 30% of revenues from S&P 500 firms were generated outside the United States (Brzenk, 2018), reflecting the growing diversity among companies and personnel within these global networks.

Future research should broaden industry coverage and increase the representation of organizations of various sizes. Conceptually, accounting for industry-specific factors that influence organizational processes and performance can uncover mechanisms and causal relationships not apparent in more generalized theories, thus enhancing the depth, relevance, and precision of our organizational theorizing. Further, given that industries have unique operational, regulatory, and competitive dynamics, broadening coverage would enable sectoral comparisons and strengthen the predictive power of our models. This need for diversity also applies to SMEs, which often operate under conditions different from those of large enterprises—such as resource constraints, localized markets, and higher risk exposure—as well as public organizations, which have different goals and accountability mechanisms compared to private entities. Expanding sampling diversity to encompass a wider range of industries and organizational sizes would prevent researchers from overemphasizing specific contexts and offer a more comprehensive understanding of organizational dynamics across different environments. This broader approach would ultimately enhance the field’s capacity for theoretical innovation and practical relevance.

#### Personnel Context

The personnel context within a sample reflects the representation of various identities and sociocultural groups within organizational workforces. This includes demographic factors, such as gender, racio-ethnicity, and age, as well as broader employment-related factors, such as education and tenure (Bell et al., 2011; Dobrow et al., 2018; Roberson, 2019). Consistent with findings in the broader diversity literature, which highlights the variability in the underlying mechanisms and outcomes across types of diversity (see Harrison, Price, & Bell, 1998; Harrison, Price, Gavin, & Florey, 2002), our results suggest that personnel diversity leads to complex variations in capabilities, behaviors, and interactions between employees. Thus, future research should account for the features of personnel contexts within organizations to better capture these dynamics.

Although demographic and employment variables are often used as moderators in research models, treating them as contexts can offer deeper insights into how these factors influence organizational outcomes. For example, capturing relational demography—or the comparative demographic characteristics of people within a given context along specific dimensions (Riordan, 2000), such as gender or tenure—can help researchers understand how different types and levels of heterogeneity influence relational dynamics and, subsequently, organizational functioning. Similarly, recognizing potential subgroups within organizations based on employee characteristics may reveal how the alignment (or misalignment) of differences shapes behavioral patterns. Expanding the scope of sample diversity, such as incorporating non-professional workers or those engaged in more precarious work, can further enrich theories of work-related phenomena by broadening their applicability. Additionally, demographic and employment factors may interact with geographic and organizational contexts to shape psychological, behavioral, and organizational outcomes. Thus, researchers must consider the personnel contexts of their samples to provide insights that are relevant across a broader range of workforces.

### Why Sample Diversity Matters for Theory and Practice

While organizational research can develop and test generalizable theories within WEIRD contexts, such as organizations readily accessible to researchers, the “WEIRD people problem” in organizational research applies within certain boundary conditions. The challenge is that until we know when these conditions hold and which theories vary cross-culturally, it is difficult to evaluate current findings fully. For example, a leadership researcher in a WEIRD country might consider that her findings may vary based on the sample population. If she aims to study leadership broadly rather than just “WEIRD leadership,” she should test theories across diverse samples. That is, theories developed based on WEIRD samples, such as those from Western countries, might not generalize to non-WEIRD contexts, such as more collectivist cultures. By studying more varied samples, researchers can either confirm the universality of a theory or identify its cultural boundaries, leading to more robust and globally applicable theories. For example, Hofstede’s cultural dimensions theory, initially based on studies of IBM employees, was later refined through research across multiple countries, providing a more nuanced understanding of cultural differences in organizational behavior. However, even for researchers focusing on a specific WEIRD context—such as U.S. leadership styles—a systematic assessment of where samples originate would be valuable. We should not ignore the distribution of sample origins, just as we cannot overlook the skewness and kurtosis in data analysis. While a skewed distribution might be acceptable, researchers need to know the extent of the skew to select the best models.

Developing theories outside of WEIRD settings and giving those developments a platform can lead to the discovery of new constructs and frameworks. Over 40 years ago, the field of organizational research experienced a period of theoretical advancement when Japanese management theories were integrated into American and European research and organizations (Keys & Miller, 1984; Pascale & Athos, 1981). Yet, such cross-cultural adaptations are scarce, likely constrained by the homogeneity of research samples and relevant stakeholders. Incorporating diverse samples and theories can reveal organizational phenomena that are absent or less pronounced in WEIRD contexts, leading to the development of new constructs. For example, the concept of *guanxi* (a Chinese term for personal connections and networks) emerged from research in China, enriching the understanding of networking and relationship-building in organizational contexts and highlighting the limitations of Western-centric views on professional relationships.

Theories developed with diverse samples can promote more equitable and inclusive organizational theories and practices. Understanding how discrimination or bias operates in different cultural contexts can lead to more effective strategies for promoting diversity and inclusion in global organizations. For example, research on gender dynamics in non-WEIRD contexts, such as in South Asia or the Middle East, can inform global diversity initiatives by revealing unique barriers women face in these regions and suggesting culturally appropriate interventions.

Exposure to diverse samples can stimulate creative thinking and innovation in theory development. Researchers might be inspired to integrate cultural perspectives or blend elements from various contexts to create novel theoretical frameworks. For example, combining Eastern philosophies (e.g., Confucianism, Buddhism) with Western management theories has led to the development of hybrid models of leadership and organizational behavior that incorporate both holistic and analytical approaches.

Furthermore, the impact of organizational research is not limited to understanding and analyzing current organizational forms. The field also has a role in proposing alternative perspectives and innovative solutions to the challenges faced by society, organizations, and individuals. Just as engineers invent new technologies and scientists develop new medical treatments, organizational researchers should aim to create new models and interventions. Achieving this requires understanding the factors that drive innovation in a research field, with diversity being a key factor (Schimmelpfennig, Razek, Schnell, & Muthukrishna, 2021). Practically, the inclusion of diverse samples in organizational research can lead to greater creativity in theorizing, enhanced problem-solving abilities, and a better understanding of global challenges.

### Increasing Sample Diversity

It is important not to confuse a constrained understanding of generalizability with the notion that generalizability should be the primary goal of all research efforts in organizational science. In many cases, research projects are focused on highly specific applied settings, which can enhance the validity of findings when samples are drawn primarily from these settings. Testing in well-known contexts can also increase internal validity by ensuring the causal homogeneity of the tested association (James, 1983). Yet, while initial testing within familiar contexts can establish a solid foundation for future research, theories that are repeatedly tested in the same narrow contexts risk reinforcing their own boundary conditions and limitations, ultimately obscuring their broader applicability. Western contexts should not be assumed as a universal benchmark, and broader sampling is needed to improve the ecological validity of research findings.

When considering the need for sample diversity, researchers must navigate a tradeoff between convenience sampling and data quality. Researchers often sample in contexts they know well, including the subject pools of their academic institutions, organizations with which they have developed relationships, or established online panel providers. One benefit of such sampling approaches is the ability to evaluate better data quality, which can be more challenging in unfamiliar or less controlled contexts. However, because convenience samples lack randomization and often do not represent the broader population, there is a higher risk of systematic differences that can lead to biased results. Thus, while ensuring data validity, reliability, and practicality is important, researchers must also take deliberate steps to foster greater sample diversity. By expanding their sampling strategies, researchers can enhance the representativeness of their findings, leading to more robust and globally relevant theories.

#### Reporting practices

Understanding the generalizability of scientific findings—knowing the extent to which a given finding applies to other contexts or the general population—is a fundamental aspect of all empirical research. At its core, understanding constraints on generalizability helps delineate the contexts in which a finding is most relevant or effective. Although transparency and clear communication about the generalizability of scientific findings are critical for accurate interpretation and application, current reporting practices in the organizational sciences often obscure these limitations. This lack of clarity is particularly concerning given that 86% of articles predominantly come from North America and Europe, which implicitly establishes a normative prototype (Medin, Ojalehto, Marin, & Bang, 2017). This prototype assumes that findings from WEIRD contexts are the standard, thereby labeling research outside such contexts as “cross-cultural.” This is also a tendency to test whether findings from non-Western countries apply to WEIRD contexts, further reinforcing this normative bias. A review of psychological research shows that articles featuring samples from “less WEIRD” contexts are seven times more likely to name the sample country in the title than articles with samples from “WEIRD” contexts (Kahalon, Klein, Ksenofontov, Ullrich, & Wright, 2022). While this may be attributable to various factors, there is little scientific justification for why certain countries appear more often in titles, especially in journals that do not explicitly constrain their reach to specific contexts (e.g., *American Journal of Management*).

**Figure 5:**
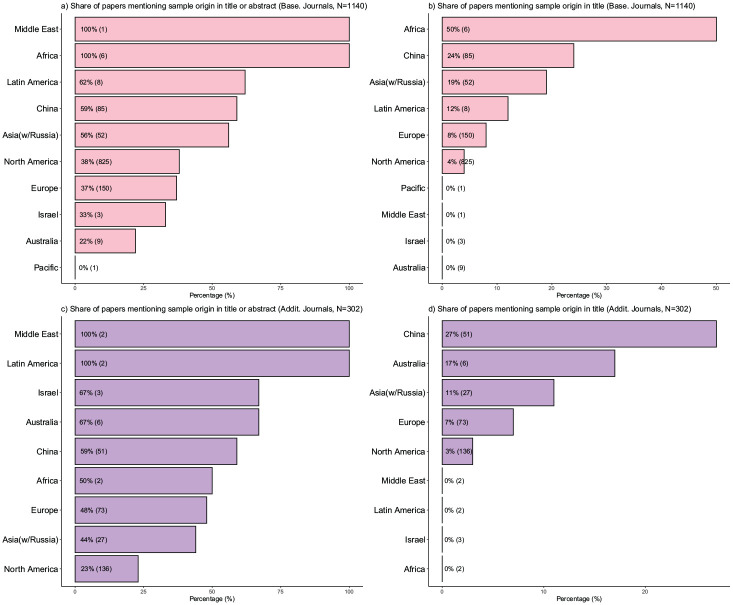
Share of Papers Mentioning Sample Origin in the Title and Abstract *Note.* Panel (a) plots the share of articles that mention the sample country in the title or the abstract. Panel (b) plots the share of articles that mention the sample country in the title. Both panels group the results by sample region and report the % of articles that mention the sample and the absolute number of articles with samples from that region. The results for both panels show a highly skewed distribution. Articles with samples originating from Africa, Latin America, China, or Asia are much more likely to report the sample country in the title and/or abstract. The results are similar in the articles from the additional journals (panels c and d), with the exception that papers with Australian samples have higher transparency about the sample origin.

It is important to recognize that although a lack of clarity regarding sample context is not an inherently fatal flaw, it does pose a challenge for readers attempting to assess the relevance of the findings in their specific contexts. A more effective and transparent approach would be to clearly communicate the sample context early in the paper, such as in the abstract. Doing so would assist other scholars in discerning the contexts in which these findings are applicable or not, thus enhancing the utility and comprehension. Our review shows that very few articles state the country of origin in the title (8%), and only a minority include it in the abstract or title (31%). In some cases (13%), it was difficult or even impossible to determine where the data was collected, which complicates understanding how the sample context might influence the findings. Figure 5 illustrates a notable skew (across both baseline and additional journals) in the proportion of papers, grouped by regions, that specify the sample country either in the title or abstract (panel a) and in the title (panel b).

The association between the sample region and the likelihood of reporting the sample country in the title or abstract is clear: Samples originating from North America, Europe, and Australia are less likely to be mentioned early in the paper. Although this may reflect the relative rarity of sample countries from less WEIRD regions, it is also notable that studies published in high-impact outlets are more likely to highlight the sample country in their title or abstract, representing a systematic bias in the field. Whereas opinions around best practices concerning reporting standards may differ, such practices should not vary based on sample origin. Importantly, we did not find that articles with fewer WEIRD samples received less scientific attention (Abramo, D’Angelo, & Di Costa, 2016; Kahalon et al., 2022). Specifically, articles based on samples from countries with lower CFST scores (i.e., the United States or countries culturally close to the United States) did not garner more citations than studies from other countries (see Online Appendix 6). Such consistency in scientific attention, regardless of sample country, should encourage researchers to sample from different countries.

#### Facilitating author diversity

Our review also revealed a clear association between author diversity and sample diversity. As summarized in Online Appendix 7, the articles in the baseline journals showed a slight bias toward U.S.-based authors, who accounted for 61.3% of the authorship. In contrast, 24.4% of authors were based in Europe, but regardless of author location, U.S. samples were still predominant. Authors from less WEIRD regions were significantly underrepresented in the reviewed articles. Specifically, authors from Asian countries comprised only 6.5% (with Russia included) and 4.8% from China. Representation from Africa (0.1%), Latin America (including the Caribbean) (0.3%), and the Middle East (0.1%) was minimal, despite these regions comprising about 29% of the global population and contributing 8% of the world’s GDP. Similar patterns were observed in additional journals, although the share of U.S.-based authors was smaller (46.4%), primarily offset by an increase in authors affiliated with institutions in China (11.9%) and Australia (6%). Reflecting social networks within the management field, authors from less WEIRD contexts often drew samples from their home countries (see Figure 7 in Online Appendix 7). This finding suggests that increasing sample diversity requires a parallel effort to increase author diversity.

### Practical Implications

Our review has several practical implications for the management field. Specifically, it illuminates actions for authors, editors, and reviewers to mitigate sampling biases within the literature and increase sample diversity. In Table 3, we offer several recommendations for doing so and subsequently enhancing the robustness, relevance, and generalizability of organizational studies.

**Table 3:** Guidelines for Alleviating Lack of Sample Diversity

**For Authors**	Declare sample origin Generalization awareness Reduce sampling based on accessibility Exploit existing diversity in samples	Specify the origin and the context of the sample. Discuss whether the findings can be generalized to other settings and explain why. Use representative or stratified samples and try to replicate studies in other contexts for testing. Also, seek collaborations with authors from different contexts and draw on literature from less WEIRD journals. Use existing sample diversity to explore cross-contextual differences and increase the robustness of findings.
**For Editors and Reviewers**	Normalize samples from novel contexts Reduce barriers for authors from less WEIRD contexts Diversify editorial teams Facilitate coordination of multisite studies	Encourage authors to not only generalize to but also from less WEIRD contexts given that sample diversity represents a contribution to methodological rigor and theoretical generalizability. Encourage submissions from authors in less WEIRD contexts and facilitate access to paper development workshops and other resources. Develop and invite associate/consulting editors from less WEIRD countries. Incentivize and facilitate multisite studies for collaborative research projects across different contexts, such as organizations or industries.

For authors, it is essential to state the origin and context of their samples clearly. Doing so enhances transparency and enables readers to better assess the applicability and relevance of the research findings across different contexts. Additionally, authors should explicitly discuss how their findings may generalize to other settings, providing explanations for their conclusions. This practice fosters critical reflection on the boundaries of the research and promotes cautious interpretation of results, avoiding overgeneralization. Authors are also encouraged to minimize reliance on convenience sampling by using more representative or stratified samples and by aiming to replicate studies in diverse contexts. Collaborating with researchers from diverse backgrounds and engaging with literature from less WEIRD journals can help ensure that findings are not skewed by accessibility biases and reflect a broader range of contexts, enhancing their validity and reliability. Furthermore, authors should take advantage of the inherent diversity within existing samples to explore cross-contextual differences, thereby increasing the robustness of their conclusions. This approach leverages available data to generate more nuanced insights and strengthens the generalizability of findings across different settings.

For editors and reviewers, normalizing the inclusion of samples from novel contexts and encouraging authors to generalize not only to but also from less WEIRD contexts is essential. Emphasizing sample diversity can enhance methodological rigor and theoretical generalizability, shifting the focus toward valuing research that incorporates a wide range of contexts. This promotes a more inclusive approach to organizational research. To reduce barriers for authors from less WEIRD contexts, editors should encourage submissions from underrepresented regions and provide access to paper development workshops and other resources. These efforts foster a more inclusive research environment, supporting scholars from diverse backgrounds and increasing the diversity of published research. Diversifying editorial teams by appointing associate or consulting editors from less WEIRD countries can also provide broader perspectives, enhancing the review process and ensuring that diverse contexts are represented and valued in published research. Additionally, editors can facilitate the coordination of multisite studies by incentivizing and supporting collaborative research projects across different contexts, such as various organizations, industries, or regions. This enhances the generalizability of research findings and fosters collaborative efforts that bring together diverse perspectives and expertise.

Overall, implementing these practical guidelines can significantly enhance the quality and impact of research in the field. By clearly stating sample origins, acknowledging limitations in generalization, reducing reliance on convenient sampling, and leveraging existing diversity within their data, authors can produce more robust and relevant research. Editors and reviewers play a pivotal role by normalizing the use of diverse samples, lowering barriers for underrepresented authors, diversifying editorial teams, and supporting multisite studies. Together, these efforts will help build a more inclusive and comprehensive body of organizational research that better reflects the global diversity of organizational contexts.

### Limitations

One key limitation of our review is the selection of journals for inclusion. We focused on the leading empirical journals in general management, a choice that likely influenced the findings. We selected these journals because they are among the most prestigious journals in the field and play a significant role in shaping hiring and tenure decisions worldwide. To provide additional context, we also reviewed papers from six additional journals, which revealed some variation in sample diversity across different areas in the field and journals. Still, it remains plausible that journals outside of North America may feature fewer U.S. samples. To gain a more comprehensive understanding of sample patterns, future research should include a broader range of journals from various regions.

Our review also faces limitations associated with coding consistency across the various dimensions of sample diversity. In some cases, it was not feasible to code for certain sample contexts due to differences in the units of analysis used in the studies. For instance, not all studies focused on organizations, making it impractical to code for organization size for those cases. Similarly, some research did not involve human participants, which prevented us from coding for participant types in those cases. Consequently, our coding for certain variables—specifically organization sector, organization size, and participant types—was restricted to a subset of papers that employed primary data collection.

## Conclusion

While the antecedents and outcomes of diversity have become increasingly important in various aspects of organizational research, such as team performance or hiring practices (Roberson, 2019), the implications of a lack of diversity in research samples remain underexplored. Importantly, diverse sample contexts are not necessary for each research project. Some research questions are best addressed using homogenous samples that accurately represent the specific context under investigation (e.g., see James, 1983, on “causal homogeneity”). Addressing the lack of sample diversity should be viewed as a collective responsibility within the broader field of organizational science rather than a task for individual researchers. Organizational research is a cumulative endeavor, building on previously published work, and is often shaped by path dependence. As a field, we should strive to ensure that research represents a variety of contexts and that findings are appropriately contextualized. This approach would allow us to better capture the diversity in organizational and managerial practices and reduce the over-reliance on evidence or theories from WEIRD countries as a default reference point for knowledge creation.

This review will likely be of broad interest to organizational research across various subfields. By highlighting the widespread lack of sample diversity, we draw attention to empirical limitations that are particularly relevant in areas like international business, cross-cultural management, human resource management, and organizational behavior. However, sample diversity is a crucial issue for all areas of organizational research. The insights from this review can help guide researchers toward more inclusive and representative sampling practices in theory-building and empirical analysis. By doing so, we can enhance the robustness and relevance of organizational research, ultimately preventing the implementation of misinformed policies and practices within organizational settings and broader societal contexts.
